# Attitudes towards a maintenance (-agonist) treatment approach in high-dose benzodiazepine-dependent patients: a qualitative study

**DOI:** 10.1186/s12954-015-0090-x

**Published:** 2016-01-08

**Authors:** Michael Liebrenz, Marcel Schneider, Anna Buadze, Marie-Therese Gehring, Anish Dube, Carlo Caflisch

**Affiliations:** Department of Forensic Psychiatry, Institute of Legal Medicine, University of Bern, Bern, Switzerland; Department of Surgery, Division of Visceral and Transplantation Surgery, University Hospital Zurich, Zurich, Switzerland; Department of Psychiatry, Psychotherapy and Psychosomatics, Psychiatric Hospital, University of Zurich, Zurich, Switzerland; Ulmenhof, Sozialtherapie, Ottenbach, Switzerland; University of Pennsylvania Health System, Philadelphia, USA

**Keywords:** Benzodiazepine dependence, Maintenance treatment, Attitudes, Withdrawal, Qualitative, Explorative, Interview

## Abstract

**Background:**

High-dose benzodiazepine dependence constitutes a major clinical concern. Although withdrawal treatment is recommended, it is unsuccessful for a significant proportion of affected patients. More recently, a benzodiazepine maintenance approach has been suggested as an alternative for patients’ failing discontinuation treatment. While there is some data supporting its effectiveness, patients’ perceptions of such an intervention have not been investigated.

**Methods:**

An exploratory qualitative study was conducted among a sample of 41 high-dose benzodiazepine (BZD)-dependent patients, with long-term use defined as doses equivalent to more than 40 mg diazepam per day and/or otherwise problematic use, such as mixing substances, dose escalation, recreational use, or obtainment by illegal means. A qualitative content analysis approach was used to evaluate findings.

**Results:**

Participants generally favored a treatment discontinuation approach with abstinence from BZD as its ultimate aim, despite repeated failed attempts at withdrawal. A maintenance treatment approach with continued prescription of a slow-onset, long-acting agonist was viewed ambivalently, with responses ranging from positive and welcoming to rejection. Three overlapping themes of maintenance treatment were identified: “Only if I can try to discontinue…and please don’t call it that,” “More stability and less criminal activity…and that is why I would try it,” and “No cure, no brain and no flash…and thus, just for everybody else!”

**Conclusions:**

Some patients experienced slow-onset, long-acting BZDs as having stabilized their symptoms and viewed these BZDs as having helped avoid uncontrolled withdrawal and abstain from criminal activity. We therefore encourage clinicians to consider treatment alternatives if discontinuation strategies fail.

## Background

High-dose benzodiazepine (BZD) dependence is an increasingly recognized clinical problem, and there is ongoing debate about its definition [1, 2] and optimal treatment strategies [3–6]. This form of dependence is not confined to users who exceed a set amount of diazepam equivalents, but is typically found in patients who have a high-dose, long-term and/or otherwise problematic use, such as mixing BZDs, escalating dosage, using BZDs for recreational purposes, obtaining them by illegal means, and/or experiencing negative social consequences [1, 3]. Although there is some evidence supporting the use of flumazenil infusions when treating this group [7–9], current guidelines favor a gradual discontinuation, with complete abstinence from BZDs as its eventual aim, irrespective of the abuse patterns mentioned above [10, 11].

While types of intervention differ (e.g., gradual BZD reduction, with a long or short half-life BZD, switching to non-BZD anxiolytics, or prescribing adjunctive medications such as antidepressants or anticonvulsants), evidence exists that high-dose-dependent individuals, in particular, are not very successful in completing the withdrawal treatment or in abstaining from BZD use in the long term [1, 12–14]. Alternatively, some authors suggest the investigation of a long-term maintenance or agonist substitution approach with a slow-onset and long-acting (= long half-life) BZD for patients who wish to discontinue high-dose BZD use but find themselves unable to abstain completely [3, 15]. There is now emerging scientific evidence that a medical intervention approach, successfully used for years in the treatment of opioid dependence [16–18], might also have a potential role in high-dose BZD dependence [2, 19]. A recent open, naturalistic study was conducted among patients who were enrolled in a methadone maintenance treatment program and had a comorbid BZD dependence. The study compared clonazepam detoxification and clonazepam maintenance treatment and found that 78.8 % of patients in the maintenance group refrained from abusing additional BZDs after stabilization and that this rate remained constant for more than an entire year. The authors therefore concluded that a specific group of high-risk patients with long-term heroin and BZD dependence and multiple attempts at BZD abstinence might fare better with a maintenance treatment approach [20].

In previous articles, we reported reasons for the initial and continued use of benzodiazepines in high doses, procurement strategies, and factors that motivate high-dose benzodiazepine-dependent individuals to stop taking these medications and on how they experienced withdrawal [14, 21]. Here, we expand this work by focusing on the perceptions of this group towards BZD discontinuation treatment versus a BZD maintenance or substitution treatment approach. This is important, since it is known that patients generally expect to improve as a result of treatment and that they are significantly more likely to discontinue treatment when their treating professionals’ assessments of appropriate treatment do not meet their expectations of helpfulness [22, 23]. In studies of opioid maintenance for example, it was reported that current opioid users “formulate shorter-term goals that do not necessarily equate with complete heroin abstinence” and use “short-term methadone episodes as self-prescribed attempts at risk reduction, and as pilot tests while considering or anticipating entering treatment to quit the use of illicit drugs” [24].

Patients’ subjective views are of clinical importance because past research indicates that individuals should be presented with a variety of treatment alternatives, rather than simply being informed about what is obtainable or easiest; in addition, this prior research has found interventions to be most beneficial when patients are prepared for what to expect of the “process of care” (e.g., description of the therapeutic setting in psychotherapy or discomfort of pharmacological intervention) and of “outcome” (e.g., clinical improvement or symptom reduction) [22, 25, 26].

Thus, the objective of the current qualitative study was to understand both the core convictions and beliefs surrounding a benzodiazepine maintenance (-agonist) treatment approach, as well as participants’ openness to an alternative to the current standard of care withdrawal treatment with complete abstinence as its aim.

## Methods

### Design

An exploratory qualitative study was conducted to investigate the perceptions and views of high-dose BZD-dependent patients on a BZD maintenance or substitution treatment strategy.

### Research sites

This study was conducted at two different sites of the Psychiatric University Hospital Zurich. The participants who were recruited at the outpatient service were interviewed at that location, while participants who were in inpatient treatment at the time were interviewed in the hospital. At both sites, interviews were conducted outside the regular treatment setting, in private offices, and in an atmosphere that permitted the participants to freely and fully express their own views and perceptions.

### Instrument development

This choice of framework guided our decision to include key areas for exploration in line with the study’s aims. Study domains were discussed and agreed upon in preparation for the study, and after an extensive literature review, a topic guide was developed that provided a flexible interview framework to explore beliefs and perceptions. Examples of interview questions surrounding a BZD maintenance or substitution treatment strategy included: “How would you feel about a substitution (a replacement) for benzodiazepines? Like, for example, heroin, that gets substituted with methadone?” Furthermore, we gave specific examples for slow-onset, long-acting benzodiazepines usually by mentioning specific brand names. We aimed to make it understandable for each individual participant that (a) a maintenance treatment intervention would not mean that they would have to completely withdraw from benzodiazepines and could potentially be on this substance for a long time and (b) that they would be switched over to a different drug formulation that would require them to take this substance less frequently, create less sedation, and result in fewer euphorigenic effects (“no flash”) but (c) would still belong to the class of benzodiazepines.

In addition, we allowed themes and motifs identified in earlier interviews to be explored in those that followed and combined the principles of maximum variation and complexity reduction to simultaneously widen the scope of results and the examination of previous assumptions.

### Sampling and recruitment

To achieve the aims of the qualitative study, a mixed method of purposeful sampling and saturation sampling principles was utilized. The sample was chosen to reflect diversity in a range of clinical aspects (past treatment experience, comorbidity, gender) as well as in socio-economic background (occupational status). Recruitment of participants by researchers was continued until saturation of data was reached, operationally defined as when no new themes emerged and we had tested all categories for disconfirming case variations. To achieve greater variation of themes and motifs, we recruited subjects from general treatment settings and from specialized units for the treatment of substance use disorders.

In total, 60 potential participants were contacted by the researchers in person: they were at least 18 years of age; were willing to give their written, informed consent; and suffered from a high-dose BZD dependence (defined as any use for an extended period of an equivalent dose of more than 40 mg diazepam per day and/or otherwise problematic use of BZDs, such as mixing BZDs, escalating their dosage, using them for recreational purposes, or obtaining BZDs by illegal means). Exclusion criteria were defined as vastly insufficient language skills and/or acute intoxication. Each patient’s chart, including a complete biographical and psychiatric history and diagnosis according to ICD-10, was made available to us by the clinic. Nineteen individuals declined to participate, and 41 individuals who met our criteria were then interviewed between 2011 and 2012.

### Study procedure

Respondents participated in individual, face-to-face, unstructured, and in-depth interviews that lasted approximately 60–90 min and were conducted by interviewers (MG/MS), who had gathered previous experience in one-on-one qualitative procedures, as well as in treatment of substance-abusing individuals. Participation was voluntary and compensation of 5 Swiss Francs was offered, either in the form of a cash payment (in the outpatient setting) or as a gift card for the same amount (in the inpatient setting). All participants were informed of their right to end the interview at any time if they wished to. In accordance with recommended principles of conducting qualitative research, the interview began with narrative opening questions, but our topic guide provided a flexible interview framework to explore beliefs that were not spontaneously covered in participants’ initial narratives. We were careful to ask open-ended and neutrally worded questions to avoid eliciting socially desirable responses. Additionally, appropriate non-judgmental and non-leading probes (echo probe, “uh-huh” probe, “tell-me-more” probe) were used during the interviews to explore perceptions that were not covered spontaneously in patients’ initial narratives. Finally, we used summarizing prompts to ensure that participants’ meaning had been accurately depicted, especially in regard to their views on a BZD maintenance or substitution treatment approach.

### Ethics

The research protocol was reviewed and approved by Zurich’s cantonal ethics committee. All participants were assured confidentiality and provided their written, informed consent, specifically to the digital recordings of the interviews. Identifying information was removed from the transcript, which were then assigned a code number.

### Data management and analysis

Data collection and analyses were conducted simultaneously until saturation had been reached. All interviews were digitally recorded, using dictamus for iOS, and then transcribed verbatim. Transcripts were compared with the recordings by the research team and validated with patients, if necessary.

Mayring’s qualitative content analysis approach was used to evaluate findings. This framework constitutes a controlled approach for empirical and methodological qualitative analysis of texts within their context of communication, following content analytical rules and step-by-step models, without rash quantification [27]. Materials were coded using an inductive qualitative procedure [28], and the research team (ML, AB, MG, CC) held biweekly discussions on the categories that were obtained to validate ratings and achieve consensus. ML applied the final code, with confirmation of consistency through blind dual coding of transcripts with MG and CC.

## Results

Table 1 presents self-reported benzodiazepine consumption patterns, mental health problems, treatment behaviors, and attitude towards a benzodiazepine maintenance approach. Our 41 study participants had a mean age of 39.5 years ± SD 9.2 (median 39.0 years, range 21 to 65 years).

**Table 1:** Self-reported benzodiazepine consumption patterns, mental health problems, treatment behaviors, and attitude towards a benzodiazepine maintenance approach

		Number	Percent
Number of participants		41	
Gender			
	Female	10	24.4
	Male	31	75.6
Duration of use			
	Under 5 years	14	34.1
	5 to 10 years	12	29.3
	More than 10 years	14	34.1
	Could not recall	1	2.4
Age at initial benzodiazepine use			
	Under 25 years	15	36.6
	25 to 40 years	18	43.9
	Over 40 years	7	17.1
	Could not recall	1	2.4
Maximum dosage of a benzodiazepine that were ever used (expressed in diazepam equivalents)			
	Under 50 mg	14	34.1
	50 to 100 mg	14	34.1
	More than 100 mg	13	31.7
Number of inpatient discontinuation attempts			
	Less than 1	1	2.4
	1 to 3	24	58.8
	4 to 9	9	22.0
	More than 9	7	17.0
Number of comorbid psychiatric diagnosis groups except substance use disorders (ICD-10)			
	None	10	24.4
	One	15	36.6
	More than one	16	39.0
Comorbid psychiatric diagnosis groups except substance use disorders (ICD-10)			
	Schizophrenia	1	2.4
	Mood disorders	21	51.2
	Neurotic disorders	12	29.3
	Personality disorders	14	34.1
Lifetime substance use other than benzodiazepines			
	Alcohol	26	63.4
	Cannabis	15	36.6
	Heroin	28	68.3
	None	4	9.8
Past or current experience with opioid maintenance treatment (OMT)			
	Yes	25	61.0
	No	16	39.0
Employment status at the time of interview			
	Employed	12	29.3
	Not employed	11	26.8
	Retired	1	2.4
	Disability pension	16	39.0
	No data	1	2.4
Attitude towards an benzodiazepine maintenance approach			
	Rather positive	18	43.9
	Rather negative and undecided	19	46.3
	No data	4	9.7

In this explorative study, participants often used the term “substitution” in their initial narrative, primarily while referring to the substitution of benzodiazepines for other psychotropic substances like alcohol or heroin. The statement of VP22 exemplifies this perception:

“…Initially I took mostly heroin and when I wanted to sleep I just waited until the flash wore off. But when I could not fall asleep this way, then I thought, instead of taking more Heroin, I take a benzodiazepine, then I did not just save money, but it also helped better to fall asleep. O.k. with heroin you can also fall asleep, but actually out of cost considerations I switched over to benzodiazepines...”

VP 22, male, 46 years

Furthermore, participants associated “substitution” with the replacement of BZDs with a non-BZD class of agents such as antipsychotics or antidepressants, which was something they had often experienced during treatment. In addition, some participants described a long-term BZD maintenance approach with a slow-onset, long-acting BZD, when referring to previous experiences and preferences for different kinds of BZDs. Only few participants had heard of a “maintenance” approach by their treating physicians, reflecting the heterogeneity of this sample in regard to treatment duration (weeks to years) and form of intervention, ranging from abstinence-oriented benzodiazepine discontinuation approach to the more permanent prescription of slow-onset, long-acting BZDs.

Perceptions and beliefs about an agonist treatment (or “maintenance”) approach had to be elucidated using non-judgmental questions: “How would you feel about a substitution for benzodiazepines? Like, for example, heroin, that gets substituted with methadone?” Furthermore and in cases of highly knowledgeable participants, we gave more specific examples for slow-onset, long-acting benzodiazepines usually by mentioning specific brand names.

However, participants’ statements in regard to such a treatment strategy sometimes appeared to contradict their previous statements or explanations. Although we tried to clarify apparent inconsistencies using additional probes, they persisted in four instances:

“…For me, personally, that is nothing…I think that is a stupid question, but with heroin you have, but I never tried heroin, as far as I know you have a “high.” And that is something you don’t have with methadone. The “high” feeling is removed with methadone—it just eases withdrawal effects…And benzodiazepines do not make a “high,” so there is no “high” feeling, at least not with me…so I would not take (substituting drugs) since I don’t have side effects from benzodiazepines…if someone just overcomes feelings of anxiety and then does not need benzos anymore, then I think it is good, if there is such a development…but I am very happy that they are around...”

VP6, male, 30 years

One participant complained of a headache upon reaching this topic and ended the interview. But the majority of our 41 participants discussed their beliefs and assumptions on this topic, allowing us to identify several common themes. Some overlap occurred between identified themes; however, no category applied to all participants. In comparison to high-dose-dependent patients’ beliefs surrounding reasons for use and experiences of withdrawal, participants tended to have rather “strong” opinions on this subject that could be subsumed into three themes:

### Theme I: maintenance treatment—“Only if I can try to discontinue…and please don’t call it that!”

Surprisingly, in this sample of long-term and high-dose BZD-dependent participants who had undergone multiple BZD withdrawal attempts, frequent relapse, and hospital re-entries, most were ambivalent about a maintenance treatment approach with slow-onset, long-acting BZDs. While they could envision the regular intake of a prescribed BZD on a long-term basis, it became clear that they were convinced that discontinuation treatment was the right treatment strategy for them.

“…I know which route I want to take… I want to zero, with both Valium → (diazepam) and methadone… I am committed to withdrawal, no matter what happens...”

VP6, male, 30 years

“…But what I started to realize during this stay (in the hospital) is that I actually increase my dosage… Initially I want to reduce the dosage and then I want to see how that goes, but I have the goal that in the sometime in the future I can live without prescribed drugs. At least without benzodiazepines....”

VP04, female, 35 years

These views were often influenced by what participants perceived as their physician’s advice. However, even participants who were skeptical about a BZD maintenance approach felt that long-term prescriptions from their physicians for BZDs were fine as long as they could continue to focus on discontinuation in the future. They were more open towards substitution treatment with non-BZD alternatives, although many immediately pointed out perceived shortcomings of neuroleptic medications and antidepressants.

“…Then I would be dependent on something. Not of a benzo but of a benzo substitute…No, no, I think that is out of the question. Maybe during reduction, and one-two weeks after that, here and there a Remeron → (mirtazapine)…Short treatment with Seroquel → (quetiapine), if you cannot find sleep or you are nervous, that is O.K. An antipsychotic has just not the same potency like a benzodiazepine. Short-time treatment, I like, yes…but more long-term, probably not...”

VP25, male, 32 years

“…I get a headache from that (Seroquel → (quetiapine)). As a benzodiazepine substitution, it is nothing for me. Maybe to relieve craving for half an hour, but not as a benzo substitute...”

VP37, female, 24 years

A considerable margin of participants who were involved in an opioid maintenance treatment at the time of the interview were equally hesitant to embrace a BZD maintenance strategy, since they felt it would limit their freedom of movement.

“…ahmm, I already made that mistake with methadone...”

VP05, male, 32 years

### Theme II: maintenance treatment—“More stability and less criminal activity…and that is why I would try it.”

Although some participants viewed an agonist maintenance treatment approach rather cautiously, the vast majority spontaneously, and without further probing, described the possible advantages of such a strategy. Almost all had had tried (either by legal or illegal means) different BZDs with diverse pharmacokinetic properties and therefore relied on their own experiences and perceptions when making assertions, and they believed that a maintenance intervention with a slow-onset and long-acting BZD such as Xanax retard → (alprazolam), or Rivotril → (clonazepam), provided them with more stability. For example, it would allow them to take a BZD once daily, instead of several times per day.

“…Valium → (diazepam) has a constant level, It does not go up and down like Temesta → (lorazepam)…Valium → (diazepam) has a longer half-life, so I do not have to take it several times a day…My psychiatrist told me that I might just never make it to completely zero…If anxiety and panic attacks return, then I will just have to continue to take the medicine… For me, it is just important to take as little as possible, so that my short-term memory is not getting any worse and it does not turn into Alzheimer...”

VP17, male, 45 years

“…Because you can use Valium → (diazepam) as a drug that creates a level. With me, it is about that I can’t turn off at night, that I can’t sleep. I am working as electrical engineer and I was also working in the sleep, looking for solutions and finding them…My psychiatrist told me that I must get away from benzos. They have increased my dosage of Depakine → (valproatic acid) and since then I just have problems. Your life quality starts suffering. I don’t feel anything, my libido is less...”

VP39, male, 40 years

Some participants reported they had experimented with different kinds of BZDs and felt the ones that lasted longer were the most beneficial to them; this had changed their preferences and consumption patterns.

“…Seresta → (oxazepam) was the only benzo where I did not have this paradox effects (compared to Rohypnol → (flunitrazepam) and Dormicum → (midazolam)). It takes anxiety…it did not have euphoric effects on me…meanwhile, I am at the point at which I notice that Seresta → (oxazepam) in a certain dosage keeps me from consuming stronger BZDs. In any way I need to take it, in just a little higher dosage, that I do not have the craving for Dormicum → (midazolam)…I took Seresta → (oxazepam) solely for half a year, a year, but then I reduced it. It protected me from using Dormicum → (midazolam)...”

VP31, male, 43 years

“…At the moment, this is right for me (Xanax retard→, alprazolam). I do not feel craving. But I do not know how it is if I have no benzo at all, if then the lust after Dormicum → (midazolam) would return. But at the moment, I somehow do not have a desire for benzodiazepines...”

VP02, male, 38 years

Interestingly, some participants had talked to their physicians about taking longer-lasting BZDs in a regulated setting, but had received mixed responses.

“…My physician always told me that I should find another doctor a pharmacy that gives it to me (on a daily basis). You go there, frequently, get your dosage. Today, eight; tomorrow, eight. Not for a week, because it is so difficult to manage. I hide that stuff so carefully that I could not find it anymore, a supply for a week…This is all crap… There was just never a possibility in Zurich to get this stuff prescribed. Although I must have addressed this issue a hundred times…well, back to the benzodiazepines, if you should substitute them, then in a little pharmacy, where you will daily get what you need. A realistic amount for people who are dependent and something long-term to cover you...”

VP34, male, 31 years

Another widely held belief among the participants of this study was that long-term maintenance for dependent individuals would result in a closure of the black market, reduce criminality, and enable individuals to stay away from the “street.”

“…I mean I see it on the street every day—so many people are hunting after this crap. I mean then the pressure would be gone if you could get it from a doctor...”

VP02, male, 38 years

“…you should see what is going on in the Langsstrasse on Sunday, every Sunday, when most pharmacies are shut down and then there are too few benzos…and then people come from the Aargau...”

VP24, female, 22 years

### Theme III: maintenance treatment—“No cure, no brain and no flash…and thus just for everybody else”

Consensus among the more skeptical participants was that a BZD maintenance approach would leave them dependent on a psychotropic substance and would therefore have a major impact on their individual decision-making about such a treatment strategy.

“…It is just the same. It would make you addicted… you are still in the same dependency…it needed to be something that I could use in high doses and still leave me fit…if something like this would be in existence, it would certainly mash your brain...”

VP07, male, 41 years

“…I still would have the feeling that I am dependent on benzodiazepines…but I think (a substitution) could be very beneficial for other patients...”

VP12, female, 66 years

Subjects also indicated that they were concerned about long-term cognitive impairments, especially in light of what they perceived to have heard from their treating physicians; they therefore wanted more scientific data.

“…I want to live without such a substance. My physician outside always tells me that, that you will get dementia, if you take this medicine too long...”

VP29, male, 50 years

“…(My physician) warned me about it. I should be careful. This stuff makes you dependent and forgetful. I should rather drink one or two beers in the evening, instead of taking so much medicine. Out of my perspective he is right, why should I be taking so much medicine?...”

VP19, male, 33 years

Some participants expressed concern about possible interactions with concurrent somatic medications and therefore found any potential benefit difficult to assess.

“…It needs to be compatible with my HIV drugs...”

VP31, male, 43 years

Contrary to a priori expectations, some high-dose-dependent patients who were abusing short-acting and fast-onset BZDs felt uncomfortable about a long-term maintenance approach with a slow-onset, long-acting BZD because they were seeking a euphoric “high” and were afraid that maintenance would make this impossible.

“…This is what I have right now with Valium ® (diazepam). It is really stupid because it does not kick. I cannot inject anything. It is not a drug. Valium ® (diazepam) is a medicine, for me. And Dormicum ® (midazolam) is a drug. A drug has to kick in and a medicine is supposed to cover something...”

VP24, female, 22 years

“…I get Valium ® (diazepam) as a substitute. It is not the same because I am an injecting user and not someone who swallows tablets. I am an injecting tablet user. You just don't get a benzo withdrawal, but it is not this most beautiful and amazingly wonderful blue tablet that so nicely kicks in, it is just Valium ® (diazepam)…that I only take in an emergency, because Dormicum ® (midazolam) is the absolute greatest of all medicine...”

VP24, female, 22 years

Virtually, all participants believed that a maintenance strategy was important and worth suggesting to individuals they knew and perceived as highly dependent.

“…A substitution for benzos…hmm…that would be useful for people who always try and always fail and who then say they do not want that anymore. For those, it would be reasonable...”

VP27, female, 36 years

## Discussion

This explorative narrative study investigated patient perceptions and views of discontinuation treatment as well as a BZD maintenance or substitution treatment strategy. Generally, the results suggest that patients with a high-dose BZD dependence who had undertaken multiple attempts at withdrawal, in inpatient as well as outpatient settings, favored a discontinuation treatment approach with complete abstinence from BZDs as its aim, despite experiencing frequent relapse. A maintenance treatment approach with continued prescription of a slow-onset, long-acting agonist was perceived ambivalently, ranging from positive and welcoming attitudes to caution and even rejection. These findings among a sample of high-dose-dependent BZD users, with and without a comorbid opioid dependence, align with two reports about BZD-dependent patients currently in opioid maintenance treatment. One of these studies reported that 71 % of current BZD users intended to stop BZD use completely, or at least wanted to try to [29]. The other found that even patients who experience frequent detoxification failure were difficult to attract to a trial using a BZD maintenance approach [15].

We also found that participants associated the use of slow-onset, long-acting BZDs with an increase in perceived stability, reduced illegal and criminal activity, less craving for short-acting BZDs, and a change in their consumption pattern of fast-onset, short-acting BZDs. Especially, those not using BZDs solely for recreational purposes developed a preference for BZDs with a slower onset and a longer duration of action. The most important additional factor that drove their attitude towards maintenance treatment was a perceived absence of a definitive cure for BZD dependence. Side effects in the form of cognitive impairments were less important to participants, often driven by perceived warnings by physicians of an increased risk for the development of (Alzheimer) dementia [30–33].

To our knowledge, this is the first study to use qualitative methodology to explore high-dose BZD-dependent patients’ attitudes towards an agonist treatment approach. While the clinical aspects are perceived as beneficial, our findings suggest that participants’ moral convictions about dependence, and their perceptions about the beliefs of treating physicians [34], are particularly contentious issues [35].

We found that almost all participants had used a variety of BZDs, possessing diverse pharmacokinetic properties, and described times when they had either been adequately maintained by their physicians or had become more stable by illegally switching to slow-onset, long-acting BZDs with less irregularities. Recreational users who preferred short-acting, fast-onset BZDs experienced periods of lessened withdrawal and cravings.

We found that some high-dose-dependent users who specifically looked for euphoric effects opposed a maintenance approach because they feared and had experienced that their sought-after BZD “flash” was eliminated when they took slow-onset, long-acting BZDs. Interestingly, some participants were aware of the beneficial effects of slow-onset, long-acting BZDs and asked their physicians to initiate a permanent and regulated BZD disposal, essentially developing and implementing their own harm reduction strategy. The more positive views of slow-onset, long-acting BZDs among this sample of patients align with previous reports on agonist treatment interventions [15, 20], as well as with our own findings on the decreased use of additional BZDs (refrainment from additional use in 27.1 %). It also aligns with the improvement in clinical depression in patients, found by therapists (improvement in 56.3 %) who switched 48 opioid-dependent patients in opioid maintenance treatment and who had a co-occurring high-dose BZD dependence to clonazepam [36].

Patients’ skepticism of long-term pharmaceutical treatment interventions for chronic disorders is common across many medical fields, even when scientific evidence shows its effectiveness. For example, a survey of 2061 patients with type 2 diabetes who were not enrolled in insulin treatment found that they perceived the clinical efficacy of insulin as low and would blame themselves if they had to begin insulin therapy [37]. Similar findings have been reported in studies investigating individuals with hypertension who were non-adherent to antihypertensive medications: some 33 % stopped the use because they “did not like taking medications” [38]. Unfavorable attitudes towards long-term pharmaceutical interventions are even more apparent in a substance-abusing population. Despite OMTs’ proven effectiveness in reducing harm [39–41], a recent qualitative study investigating barriers to treatment entry for opioid-dependent users found, among other factors, that some patients were apprehensive about a long-term maintenance program and preferred a time-limited program and a discontinuation approach [42]. Other barriers—identified in the same study—included “real or rumored side effects, fear of withdrawal during incarceration or disinterest in adherence to the structure of treatment programs”—especially the last aspect may have, although not specifically mentioned, contributed to the unfavorable perception of some participants towards benzodiazepine maintenance treatment as well. In this context, it needs to be pointed out that 61 % of our participants had past or current experiences with OMT and may therefore have intuitively drawn parallels to such programs and their restrictions (e.g., limitations on take home dosages).

Although the comparison of BZD maintenance to an opioid maintenance treatment approach is not entirely accurate (since patients in OMT do not exhibit cognitive impairments, and side effects related to cognition and memory are one of the major limitations of a BZD agonist substitution approach [43, 44]), our explorative study shows that some high-dose BZD-dependent patients may require an alternative to a discontinuation treatment approach. While we acknowledge the possible negative consequences of such an approach, especially in individuals using multiple substances [45], we suggest that clinicians consider our findings in the context of the large group of patients who fail in abstinence-oriented treatments. We urge them to provide—or develop—some alternative guidance when they are contacted by patients who want to change their BZD consumption pattern but find themselves unable to successfully complete discontinuation programs.

We used an unstructured, topic-guided approach in line with recommendations for the use of qualitative methodology to explore patient perceptions, since little is known about this clinical subject and a semi-structured, interview-guided approach was not feasible. Although patients frequently spontaneously addressed substitution or maintenance in their initial narratives, we used some questions that contained an analogy to the treatment of heroin dependence in order to elucidate participants’ beliefs. It is possible that this could have led some of them to express unfavorable beliefs, since heroin users are a heavily stigmatized patient group. However, we believe that we can report saturated data, since we continued interviewing until no new themes emerged and all categories had been tested for disconfirming case variations.

Questions about conceptual generalizability also arise, since this study was conducted with 41 long-term, high-dose-dependent patients, the majority of whom were in inpatient treatment at the time of the interview and might therefore have preferred a discontinuation approach. However, participants who were recruited in the outpatient settings expressed similar views. An additional consideration is that 19 potential participants declined inclusion, so we are likely to have missed the personal views of individuals who felt especially sensitive about being interviewed on their BZD dependence. Although the interviews were conducted outside the treatment setting and subjects were informed that no information except suicidal ideation would be made available to the treating physicians, some participants might have responded with answers (especially in regard to their current discontinuation therapy) that they believed were being sought by the interviewer. While we acknowledge these limitations, we believe that this study adds to the scarce literature on the beliefs of long-term, high-dose BZD-dependent individuals with a diverse clinical (past treatment experience, comorbidity, gender) and socio-economic background (occupational status). Finally, the generalizability of our sample remains to be established as patients in our sample were recruited in a clinical context. The inclusion of individuals who are out of treatment would help us better understand how well our findings may apply to this population.

## Conclusions

We conclude that patients with a high-dose BZD dependence are generally in favor of a discontinuation treatment approach, despite treatment failure, because they view a maintenance treatment approach (with continuous prescription of a slow-onset long-acting agonist) ambivalently. We identified three overlapping themes that summarized patients’ perceptions on maintenance treatment: (1) “Only if I can try to discontinue…and please don’t call it that!” (2) “More stability and less criminal activity…and that is why I would try it.” and, finally, (3) “No cure, no brain and no flash…and thus just for everybody else.” Since some patients had experienced a slow-onset, long-acting BZD as stabilizing and having helped them abstain from fast-onset, slow-acting BZDs and from criminal activity, we encourage the consideration of treatment alternatives if discontinuation strategies fail. Future research needs to address the important clinical questions of side effects, focusing on the extent of cognitive impairments in a BZD-maintained population.
